# Productive and Economic Responses in Grazing Dairy Cows to Grain Supplementation on Family Farms in the South of Brazil

**DOI:** 10.3390/ani4030463

**Published:** 2014-07-17

**Authors:** Luiz Carlos Pinheiro Machado Filho, Leandro Martins D’Ávila, Daniele Cristina da Silva Kazama, Lauana Luiza Bento, Shirley Kuhnen

**Affiliations:** 1 Lab of Applied Ethology and Animal Welfare (LETA), Departament of Zootechny and Rural Development, Federal University of Santa Catarina, Rod. Admar Gonzaga, 1346, Florianopolis, SC 88034-000, Brazil; E-Mails: davila27@gmail.com (L.M.D.); daniele.kazama@ufsc.br (D.C.S.K.); 2 Lab of Animal Biochemistry, Department of Zootechny and Rural Development, Federal University of Santa Catarina, Rod. Admar Gonzaga, 1346, Florianópolis, SC 88034-000, Brazil; E-Mails: lauana@zootecnista.com.br (L.L.B.); shirley.kuhnen@ufsc.br (S.K.)

**Keywords:** livestock sustainability, grazing behavior, milk composition, Voisin’s Rational Grazing

## Abstract

**Simple Summary:**

In the South of Brazil, as in many regions where dairy production is pasture-based, the use of concentrate to supplement cattle diet frequently does not follow technical guidelines. This may result in inefficient management, with increased cost of production and lower pasture intake. In this study, small amounts of an energy supplement proved to be more economically efficient than a high protein commercial concentrate, despite a decrease in milk productivity. The cows were able to compensate for the lower levels of protein in the supplement with selective grazing for high protein plants. The quality of the milk was unaffected by the treatment.

**Abstract:**

Pasture-based dairy production has been a major source of income for most family farms in the south of Brazil. Increasing milk prices have spurred an increase in grain supplementation, which has been poorly implemented, resulting in low levels of efficiency. To evaluate the consequences of supplementation on milk production and composition, grazing behavior and economic return, the widely used grain management system (CC-commercial concentrate, containing 21% CP, offered at 1 kg per 3.7 L of milk) was compared with an energy supplement (GC-ground corn, with 9.5% CP, offered at 0.4% of live weight). Ten Holstein cows were paired into two groups, and subjected to the two treatments in a crossover design. The cows remained in the same grazing group, and the grain supplement was offered individually at milking time and consumed completely. Each experimental period lasted 14 days, with 10 days for diet adaptation and four days for data collection; individual milk production and samples were collected to determine levels of fat, protein, lactose, carotenoids, vitamin A and N-urea. Grazing behavior was observed (scans every 5 min) in the first 4 h after the morning milking, and chemical composition of hand plucked samples of forage were measured. The cost of the supplement and profitability per treatment were calculated. Cows supplemented with GC consumed herbage with higher crude protein (CP: 16.23 *vs*. 14.62%; *p <* 0.05), had higher biting rate (44.21 *vs*. 39.54 bites/min; *p <* 0.03) and grazing time (22.20 *vs*. 20.55 scans; *p <* 0.05) than when receiving CC. There were no differences in milk composition between treatments (*p >* 0.05). However, higher concentrations of β-carotene and total carotenoids were detected in the milk of cows at 70–164 days of lactation, compared to <70 days of lactation (*p <* 0.05). Milk production was higher (13.19 *vs*. 11.59 kg/day; *p <* 0.05) when cows consumed CC, but resulted in lower profitability compared to GC (US$ 4.39 *vs*. US$ 4.83/cow per day). Our results show that higher productivity does not necessarily improve profitability. Cows receiving supplement with lower levels of protein were able to adjust their grazing behavior to meet their protein needs and this level of diet modification did not alter milk composition.

## 1. Introduction

In Western Santa Catarina State, a major dairy producing region in South America, milk production is typically pasture-based, with the use of concentrate supplementation. Santa Catarina State is the fifth largest and fastest growing milk producing state in Brazil, with about 80% of the farms based on family farming [1]. The region is comprised of family farm units, with small herds (about 20 cows), relying on milk production as their main source of income [2]. A large number of producers in the region have adopted the pasture-based system coupled with the use of supplements, and there are a growing number of ecologically-based properties, some of which have obtained organic certification. Most organic farms use Voisin’s Rational Grazing (VRG), but this system is also used to manage conventional herds [3]. We estimate that VRG is used equally by organic and semi-intensive dairy farms [4]. VRG is a rotational grazing system [5], where cows typically graze a new paddock after each milking.

Family dairy farms in Southern Brazil use concentrate or corn silage to supplement grazing, particularly during seasons of forage scarcity, such as the fall and the beginning of winter [2,4]. However, organic farms are less likely to use concentrate [3,6]. Besides feeding management based on pasture, agroecological production in Brazil, and other regions of the world, is characterized as having smaller and less productive herds. This lower level of productivity has been attributed to crossbred cows, but also to the limited use of concentrate [7,8].

The subtropical climate of the region is favorable for forage cultivation all year round, allowing the succession of tropical, subtropical and temperate species in the same grassland. The climate also allows for the establishment of multispecies permanent pasture, especially when pasture management is based on the ecology of the region. In these pastures, estimating the nutritional needs of cows with distinct productive potential and stages of lactation, is quite complex [9]. Most often, concentrate supplementation is used to compensate for possible nutritional deficiencies, without a proper evaluation by the farmer if the supplement is reducing cows’ pasture consumption, and thus increasing costs. Furthermore, organic farmers are less likely than conventional farmers to use a nutritionist for ration and feeding advice [8]. Energy may be a restricting factor in dairy cow productivity at pasture, because the energy content of forage does not generally meet the productive milk potential needs of the cows [10,11]. Even at its optimal grazing point, the pasture may have high concentrations of protein, but may be deficient in non-structural carbohydrates [12]. In this case, the forage energy is insufficient to provide the potential for milk production given by the protein concentration, limiting the productivity of cows feeding exclusively on pasture. On the other hand, milk quality is positively affected by pasture feeding; the more fresh forage the animals consume, the higher the levels of carotenoids and other anti-oxidants present in the milk [13].

There is a positive response in milk yield, energy-corrected milk (ECM), fat and protein, to increasing amounts of supplement [14]. However, an excess of grain may compromise the sustainability of dairy production as the energy costs may be unsustainable. In conventional milk production, based on feeding Total Mixed Ration (TMR, a mix of cereals and forage), it is estimated that an input of 14 kcal of fossil energy is required to produce 1 kcal of milk protein. Even with lower energy costs than the production of meat and eggs, milk production could be more efficient if production was entirely based on pasture and hay [15]. In fact, some researchers have argued that to improve environmental performance in milk production, we must: (1) reduce the use of concentrated ingredients with high environmental impact; (2) reduce the use of concentrates per kilogram of milk; and (3) reduce the surplus of nutrients, improving the flow of nutrients from the farm [16]. Pasture-based feeding is financially and environmentally economical, as it reduces the input of fossil fuel energy into the system.

Feeding management may represent an important constraint in organic dairy production. The use of low cost supplementation from food sources readily available on the property, like corn meal, is preferable, as long as it does not compromise milk quality or productivity of the system. Thus, in order to assess the productive and economic responses of dairy cows to the supplementation of pasture-based feeding with grain, two management systems were assessed: the current standard quantity of concentrate was compared to an estimated quantity of energy supplement, ground corn.

## 2. Material and Methods

### 2.1. Location

The experiment was conducted on a 16.9 ha family farm located in the western region of Santa Catarina State, Brazil (26°26'04"S and 52°49'33"W), during the spring of 2011. The experiment was carried out in a 7 ha VRG system, implemented in 2009, consisting of 65 paddocks with an average area of 1,100 m^2^ each. At the time of the experiment, the pasture was comprised predominantly of *Lolium multiflorum*, *Trifolium repens*, *Axonopus catarinensis* and *Hemartria altissima*. The paddocks were used at the optimum grazing point obeying the 1st law of rational grazing [5]. The time spent in each paddock was 24 h and the instant stocking rate was 167 AU (500 kg)/ha/day. The animals were placed in a new paddock after the morning milking.

### 2.2. Animals

Of the 24 lactating cows on the farm, 10 Holstein cows were selected and paired based on body weight, days in milk (DIM), parity, concentrate consumption and daily milk production immediately before the beginning of the experiment (2 days). A detailed description of the animals is shown in Table 1. All procedures involved in this experiment were approved by the Ethics Committee on the Use of Animals of the Federal University of Santa Catarina (UFSC), protocol number PP00673.

**Table 1:** Characteristics of the animals used in the experiment: group, animal identity (ID), body weight (BW), days in milk (DIM), parity, body condition score (BCS) and milk production two days prior to the beginning of the experiment, mean and standard errors.

Group	Animal ID	BW(Kg)	DIM	Parity	BCS	Milk Production (kg/day)
**A**	73	425	40	4	4	19.5
	74	530	80	2	3	17.1
	86	480	90	1	3	10.7
	19	360	30	1	4	14.2
	49	500	150	2	3	10.9
**Mean ± SE**		459 ± 30.1	78 ± 21.3	2 ± 0.5	3.4 ± 0.2	14.5 ± 1.7
**B**	18	440	50	1	3	14.1
	57	510	60	3	3	16.1
	54	500	120	3	3	13.3
	71	360	20	1	4	14.9
	41	440	130	1	3	12.9
**Mean ± SE**		450 ± 26.8	76 ± 21.1	1.8 ± 0.5	3.2 ± 0.2	14.3 ± 0.6

### 2.3. Experimental Design and Treatment Description

The experimental design was a crossover with two treatments, involving two experimental periods of 14 days, with 10 days for diet adaptation and four days for data collection. The treatments consisted of supplementing the animals’ feed with: (a) Commercial Concentrate (CC) supplied based on the average quantity generally used in the region [4], resulting in 1 kg of feed for each 3.7 L of milk produced; and (b) ground corn (GC) offered in the proportion of 0.4% of body weight, or an energy supplement equivalent to 15% of the estimated total daily dry matter intake. The amount of supplement per animal per day was divided into two equal portions and a portion was offered during both the morning and afternoon milking in individual troughs. Whereas the estimated composition of the pasture was 14% crude protein (CP) and 53% total digestible nutrients (TDN) [9,17], the energy was the limiting factor in achieving the productive potential of pasture nutrients. During the entire experiment all animals were together in one single herd, along with 14 other lactating cows. Therefore, pasture, water, mineral mix, shade, social group and the distance between the paddock and milking parlor were equal for all cows; the only difference between the two experimental groups was the concentrate (treatment) individually assigned to the animals.

### 2.4. Bromatological Analyses of the Diet

Analyses of dry matter (DM) and crude protein (CP) in pasture and supplements were performed according to the AOAC Official Methods of Analysis [18]. For the analysis of neutral detergent fiber (NDF), the Van Soest method was used [19]. DM, NDF, and CP contents of ground corn and commercial concentrate are shown in Table 2. It is important to note that, based on our analysis, the CP level of the CC was 21.28%, which is greater than the level declared on the label of the concentrate (18%).

**Table 2:** Chemical composition of the commercial concentrate (CC) and ground corn (GC) used for animals.

Variables	CC	GC
Dry matter (%)	89.27	87.75
Neutral Detergent Fiber (%)	30.30	11.94
Crude Protein (%)	21.28	9.48

### 2.5. Behavioral Observations and Data Collection

Milk production was measured on the 12th and 14th day of each experimental period, in the morning and afternoon milking. Milk samples were collected and homogenized to compose one sample per animal per period and analyzed for fat, protein, lactose, total solids, urea-N, somatic cell count (SCC), total carotenoids, lutein, zeaxanthin, b-carotene and vitamin A.

On the 11th and 13th day of each experimental period, grazing behavior was observed, and pasture samples were collected after the morning milking. Pasture samples were collected using the grazing simulation technique [20]. Three sub-samples were taken per animal on each day of observation in the first hour, at 40 min intervals, resulting in two samples per animal per period (one amalgamated sample per observation day).

Grazing behavior of the experimental cows was also recorded on the 11th and 13th day in the morning (8:00 am–12:00 pm). Behaviors were recorded every 5 min using the instantaneous scan sampling technique [21,22]. Following the definitions adopted by the Laboratory of Applied Ethology and Animal Welfare (LETA) of the Federal University of Santa Catarina, observed behaviors included: grazing (animal with the mouth below or at the level of forage or grabbing forage, either stationary or moving forward), ruminating (animal chewing with lateral jaw movements with the head at the same level or above its body, lying or standing), walking (animal moving, with the head above the superior level of forage), drinking (animal with the lips immersed in water, with neck movements indicating water ingestion), idle (animal still, not engaged in any of the behaviors described above, lying or standing), and others (any other behavior not described above) [23].

Animals were watched continuously for four h by two observers. The observers were trained at the beginning of the experiment to ensure inter-observer reliability [21,22] and were located at least 15 m distance from the cows to avoid interference during the observation periods. Observers alternated themselves the groups observed in the two observation days. Animals were identified by ear tags and coat color. For data analysis, observation of the grazing behavior was divided into two periods of two hours each; period 1 refers to the first two hours after morning milking (8–10 am), and period 2 was from 10 am–12 pm. The biting rate was also recorded; biting was counted for 30 s three times over three 30 min intervals after the first hour of observation, totaling 9 records per animal. The average of these 9 records was used as the biting rate for each animal.

### 2.6. Milk Composition Analysis

Analyses of total solids, protein, fat, lactose and urea-N were conducted using infrared method [24] while the flow cytometry method was used to determine SCC [25]. For carotenoids and vitamin A, the methodology described by Hulshof *et al*. [26] was used. Extraction was performed using ammonium hydroxide and ethanol (2.5:6 *v/v*), followed by the addition of ethyl ether (0.0025% BHT) and petroleum ether (1:1 *v/v*). The samples were then centrifuged (4,000 rpm, 4 min), and the supernatant evaporated under N_2_ flow at 40 °C. The residue was saponified (5% KOH for 3 h at 37 °C, 200 rpm) and re-extracted with hexane. Aliquots of the concentrated sample were injected into a liquid chromatograph (Shimzadu LC-10A), equipped with a C18 reverse-phase column (Vydac218TP54, 250 mm × 4.6 mm × 5 µm), protected by a 5 µm C18 reverse-phase guard column (Vydac218GK54), and a UV-visible detector operating at 450 and 392 for carotenoids and vitamin A, respectively. The eluent was MeOH:CH_3_CN (90:10, *v/v*, 1 mL/min) and the identification of the compounds of interest was performed using retention times of standard compounds (Sigma-Aldrich, St. Louis, MO, USA).

### 2.7. Statistical Analyses

Statistical analyses were performed using the mixed procedure from SAS statistical package version 9.0, considering the model:

Y_ijk_ = µ + P_i_ + T_j_ + e_ijk_

where, Y_ijk_ is the value of the dependent variable, µ the average value, P_i_ the effect of period, T_j_ the treatment effect, and e_ijk_ the random error N (0, s^2^). The means of each treatment were compared using Tukey’s test, considering a 5% significance level.

## 3. Results and Discussion

The analysis of pasture samples collected through the grazing simulation technique (Table 3) show a higher concentration of crude protein in the diet selected by cows given the GC treatment (*p <* 0.05), the supplement with lower protein levels (Table 2). These results suggest that grazing cows can select pasture with different chemical composition to compensate for a nutrient shortage in the diet offered. In the present study, cows eating ground corn were consuming a smaller amount of protein due to both a lower concentration of protein in the supplement, as well as smaller quantity of the supplement. Previous research has demonstrated the ability of ruminants to modify one or more components of their ingestive behavior in order to minimize unfavorable feeding conditions and achieve the nutritional needs for maintenance and production [27,28].

Considering the experimental design, where all animals were given both treatments, it is interesting to note that animals were able to select a higher CP pasture diet when the supplement was poor in the nutrient. Combining corn—or any other energy supplement—with pasture that contains a significant amount of legumes would likely be a sustainable strategy as the protein in the pasture can fulfill the nutritional needs of lactating cows [11]. Relying on foraged sources of protein, such as white clover, to feed cattle can also reduce greenhouse gas emissions from pasture-based milk production, thus lowering the carbon footprint [29].

**Table 3:** Dry matter (DM), crude protein (CP) and Neutral Detergent Fiber (NDF) concentrations in pasture samples collectedby thegrazingsimulation technique with cows fed either commercial concentrate (CC) or ground corn (GC).

Variables	Treatments			
	CC	GC	SE ^1^	P ^2^
DM (%)	27.62	28.69	0.523	0.19
NDF (%)	61.69	60.80	0.891	0.31
CP (%)	14.62	16.23	0.461	0.05

^1^ SE = Standard error; ^2^ P = Probability.

The grazing frequency, recorded in 2 h periods, and the biting rate per min are shown in Table 4. The frequency of grazing was similar between treatments in the first observation period (8–10 am) after the morning milking, and different during the second observation period (10 am–12 pm). In this case, the animals that were fed ground corn extended their grazing into the fourth hour of observation while the CC group did not (*p <* 0.05). A higher biting rate was also detected during grazing for those animals given the GC diet (*p <* 0.05). These results suggest that, compared to those receiving ground corn, the animals that were fed the commercial concentrate reached a level of satiety more quickly, adjusting their grazing behavior to the condition of concentrate supplementation [11]. Flexibility in grazing behavior to achieve the same intake has been reported in other studies; different genotypes may adjust their ingestive behavior to achieve the same intake in a grazing situation [30].

**Table 4:** Grazing frequency (5 min interval) in two observation periods (Period 1, from 8 to 10 am; Period 2, from 10 am to 12 pm) and average biting rate of cows given either commercial concentrate (CC) or ground corn (GC).

Variables	CC		GC		SE ^1^	P ^2^
	Period 1	Period 2	Period 1	Period 2		
Grazing frequency	23.80 ª	20.55 ^c^	23.80 ª	22.20 ^b^	0.37	0.04
Biting rate (min^−1^)	39.54 ^a^		44.21 ^b^		1.19	0.03

^1^ SE = Standard Error; ^2^P = Probability. ^a,b,c^ Letters indicate statistical differences in the same row.

As the grazing frequency and biting rate suggest, the difference in DM intake from supplements may have caused differences in pasture intake due to a substitution of pasture with concentrate. Lactating cows reduce their grazing time with an increase in supplements from 0 to 3 or 6 kg of concentrate per day [14]. Likewise, Bargo *et al*. [31] found a reduction in grazing time by 12 min/day per kilogram of concentrate compared with a non-supplemented diet; however, in their study there was no change in biting rate (58 bites/min) or bite mass (0.47 g of DM/bite) related to supplementation. The authors suggest a substitution rate ranging from 0.2 to 0.6 kg of pasture DM per DM kg of supplement. In the case of our study, it was not possible to measure pasture intake; however, we can infer that there was a reduction in the intake of pasture due to shorter grazing frequency in the second observation period, which was associated with a lower biting rate.

The total production of milk, fat, protein, lactose and total solids was higher in cows given commercial concentrate (*p <* 0.05, Table 5). However, the contents of fat, protein, lactose and total solids of milk were not affected by treatment (*p >* 0.05). The higher amounts of milk production from animals receiving commercial concentrate can be related to a higher intake of TDN in this treatment. Beyond the differences in supplement composition, a greater portion of CC was fed to lactating cows than the GC portion. Thus, the expected higher milk production from cows in CC treatments is in accordance with studies showing increased production of grazing cows with greater amounts of supplements [11,32].

**Table 5:** Average production of 3.5% fat corrected milk (FCM) and milk composition of cows on pasture supplemented with commercial concentrate (CC) or ground corn (GC).

Variables	Treatments		SE ^1^	P ^2^
	CC	GC		
3.5% FCM, kg/d	13.19	11.59	0.818	0.01
Fat%	3.29	3.24	0.188	0.69
Protein%	2.95	2.94	0.083	0.88
Lactose%	4.60	4.57	0.057	0.19
Total Solids%	11.78	11.67	0.283	0.45
Fat kg/d	0.45	0.39	0.029	0.02
Protein kg/d	0.40	0.35	0.022	0.01
Lactose kg/d	0.64	0.55	0.373	0.01
Total solids kg/d	1.61	1.41	0.092	0.01
SCC^3^ Log	2.00	1.81	0.266	0.29
N-urea mg/dL	14.46	13.41	0.589	0.25

^1^ SE = Standard Error; ^2^ P = Probability; ^3^ SCC log = log_10_ of somatic cell counts.

The somatic cell count (SCC) values did not differ between groups (*p <* 0.05) and all samples met Brazilian legal standards, thus indicating the health of the herd. Similarly, the concentration of N-urea was not affected by treatments (*p <* 0.05) and ranged between 10 and 15 mg dL^−1^, indicating appropriate levels of dietary CP [33].

We would expect higher levels of carotenoids in the milk from cows fed ground corn as compared to concentrate [34]. Likewise, a higher intake of pasture for animals of the GC group would also be expected. Pasture and ground corn are likely to increase the levels of vitamins and their precursors in milk. However, in this experiment, the dietary differences did not affect carotenoid levels in the milk (*p >* 0.05). An interesting result of our study is that the contents of total carotenoids, β-carotene, vitamin A and vitamin A equivalent differ between lactation periods up to 70 days and beyond 70 days, regardless of treatment (Table 6). Higher contents of total carotenoids and β-carotene (*p <* 0.05) were detected during the lactation period beyond 70 days. These results differ from those obtained by Winkelman *et al*. [35], which showed a decrease of β-carotene during lactation. Changes in the secretion of carotenoids in milk during lactation may be attributed to a decrease in milk production or to the animal’s ability to capture these pigments from blood plasma [13,36]. In our study, the results corroborate the hypothesis that a decrease in milk production that occurs in the second lactation period, and consequently the total fat produced, might result in a higher concentration of β-carotene per unit of fat at this later lactation stage. However, a specific effect on the stage of lactation has not been clearly established, since this may be linked to the seasonality of the feed.

**Table 6:** Carotenoid and Vitamin A contents (μg/g of fat) of milk produced at the initial and median phase of lactation by cows on pasture supplemented with GC or CC.

Variables	Days in Milk		
	0 to 70 Days *n* = 9	More than 70 Days *n* = 11	*P*
Total carotenoids	7.77	10.12	0.05
β-carotene	4.88	7.40	0.02
Vitamin A	7.95	9.26	0.53
Vitamin A equivalent	9.84	11.48	0.50

The concentrations of β-carotene found in this study are similar to those produced in milk from cows receiving supplementation in the northern hemisphere during the grazing season [26,37]. However, they are below the values found for animals kept exclusively on pasture in the northern hemisphere, suggesting that concentrate supplementation has a negative impact on this bioactive constituent of milk, even for cows on pasture [37,38].

The cost and revenue estimates related to supplementation and milk production were calculated and are shown in Table 7. The gross revenue of the CC group was US$ 6.07; however, based on the daily cost of feeding, animal production and sale value, the group posted a net return of US$ 4.39. For the GC group, the net return was US$ 4.83. Thus, the greater amount of milk produced by the CC treatment was not accompanied by an economic advantage for the producer and these aspects need to be considered in dairy production management. Extrapolating these values to this property with 25 lactating cows, opting for a GC supplement would result in an increase of US$ 0.44/animal per day, or US$ 332.35/month. Adopting ecologically-based practices in pasture and cattle management appears to reduce the cost of production and improve revenue. Improved economic performance has been observed in Western Santa Catarina State properties, the majority of which have adopted agroecological pasture management. It was also observed that the greatest economic return was strongly correlated with a higher rate of adoption of agroecological practices for pasture management [39].

**Table 7:** Economic impact of supplementing grazing cows with commercial concentrate (CC) or ground corn (GC). All other costs considered the same.

Variables	CC	GC
Daily supplement intake	3.22 Kg	1.78 Kg
Price of supplement (US$/kg)	US$ 0.52 ^1^	US$ 0.28
Daily cost	US$ 1.69	US$ 0.51
Daily production /cow	13.19 L	11.59 L
Milk price/L	US$ 0.46	US$ 0.46
Daily gross income from milk per cow	US$ 6.07	US$ 5.33
Daily net income from milk per cow	US$ 4.39	US$ 4.83

^1^ US$ 1.00 = R$ 1.74, in October 2011.

## 4. Conclusions

This study shows that higher milk productivity does not necessarily improve profitability. Cows with fewer supplements were able to adjust their grazing behavior to meet their protein needs, and this type of dietary modification did not alter milk composition. Future studies should focus on the adequacy of supplementation, taking into account the nutritional requirements of the cows, stage of lactation and distinct quality of pasture throughout the year, in order to improve economic efficiency without jeopardizing the quality of the milk produced.
